# Tribological Characteristics of Fibrous Polyphthalamide-Based Composites

**DOI:** 10.3390/polym16162274

**Published:** 2024-08-10

**Authors:** Yuanyi Shen, Dmitry G. Buslovich, Sergey V. Panin, Lyudmila A. Kornienko, Pavel V. Dobretsov, Yury M. Kolobov

**Affiliations:** 1 Department of Materials Science, Engineering School of Advanced Manufacturing Technologies, National Research Tomsk Polytechnic University, 634050 Tomsk, Russia; shenyy1118@mail.ru; 2 Laboratory of Nanobioengineering, Institute of Strength Physics and Materials Science of Siberian Branch of Russian Academy of Sciences, 634055 Tomsk, Russia; buslovich@ispms.ru; 3 Laboratory of Mechanics of Polymer Composite Materials, Institute of Strength Physics and Materials Science of Siberian Branch of Russian Academy of Sciences, 634055 Tomsk, Russia; rosmc@ispms.ru; 4 DKM Engineering LLC, 620075 Ekaterinburg, Russia; dpv@tpc-mac.ru (P.V.D.); kolobov.um@gmail.com (Y.M.K.)

**Keywords:** polyphthalamide, composite structure, injection molding, interface, carbon fiber, glass fiber, coefficient of friction, wear rate, tribological layer

## Abstract

The aim of this study was to investigate the tribological characteristics of commercially available high-strength polyphthalamide-based composites with great contents (30–50 wt.%) of both carbon and glass fibers in point and linear contacts against metal and ceramic counterfaces under dry friction and oil-lubricated conditions at various loads and sliding speeds. The lengths of both types of fibers were varied simultaneously with their contents while samples were fabricated from granules by injection molding. When loading PPA with 30 wt.% SCFs at an aspect ratio (AR) of 200, the ultimate tensile strength and the elastic modulus increased up to 142.7 ± 12.5 MPa and 12.9 ± 0.6 GPa, respectively. In the composites with the higher contents of reinforcing fibers PPA/40CCF and AR~1000, the ultimate tensile strength and the elastic modulus were 240 ± 3 MPa and 33.7 ± 1.9 GPa, respectively. Under the applied test conditions, a composite reinforced with 40 wt.% carbon fibers up to 100 μm long at an aspect ratio of ~1000 possessed the best both mechanical properties and tribological characteristics. One of the reasons that should be considered for improving the tribological characteristics of the composite is the fatigue wear mechanism, which is facilitated by the high filling degree, the strong interfacial adhesion, and the great aspect ratio for fibers. Under the oil-lubricated conditions, both friction coefficients and wear rates decreased, so such friction units could be implemented whenever possible. The reported data can be used as practical recommendations for applying fibrous polyphthalamide-based composites as friction unit components.

## 1. Introduction

Despite the increasing use of high-performance polymers (HPPs) currently [1,2], their high cost, stringent requirements for both equipment and processing modes, and the complexity of designing composites reinforced with long discrete fibers are among the limiting factors on the path to their widespread industrial implementation [3,4,5,6]. On the other hand, achievements in chemical science and technology enable us to produce advanced matrix materials based on more common and effective thermoplastics [7,8]. They are not inferior to HPPs in a number of functional properties, while being significantly cheaper. Such grades include (semi-)aromatic polyamide (PA), the undoubted advantage of which is a high melt flow index (MFI). So, it can be used as a matrix for the fabrication of high-strength composites reinforced with fibers up to 10 mm long in large quantities.

**Figure 1:**
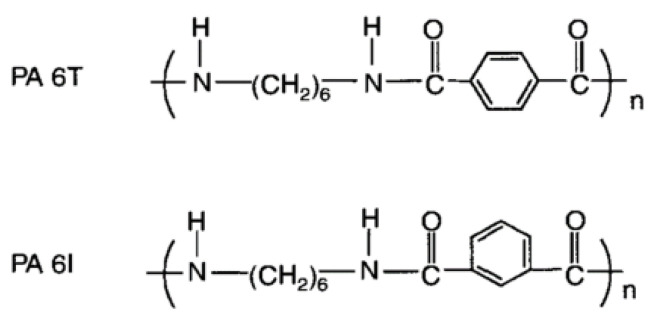
Chemical structures of PPAs synthesized from terephthalic (6T) and isophthalic (6I) acids.

Polyphthalamide (PPA) is a new type of heat-resistant semi-aromatic PA, synthesized from terephthalic or isophthalic acids [9]. Depending on the chemical composition of PA, terephthalic and isophthalic acids can be combined to produce PPAs with different chemical structures (designated as PAXT, PAX.YT, PAXI, or PAX.YI). Figure 1 shows examples of the chemical structure of PPAs synthesized by combining hexamethylene diamine with terephthalic (PA6T) or isophthalic (PA6I) acid, respectively [10]. Currently, such polymers are commercially produced by only a few of the world’s manufacturing leaders.

After grafting aromatic rings onto a polymeric molecular backbone, PPAs possess better thermal stability than aliphatic polymers (their melting points exceeding 280 °C). Another undoubted advantage of PPAs is their two times lower water absorption compared to those of both PA6 and PA66, for example.

At present, PPAs, along with highly structured polyether ketone (PEEK) and polyphenylene sulfide (PPS), constitute a family of thermoplastic polymers with great strength properties and improved heat resistance (Table 1). The high flexibility of PPA polymer chains, allowing to load up to (40–50) wt.% fibrous fillers (primarily carbon fibers (CFs) as well as glass fibers (GFs)) into their composites, enables us to design advanced materials with strength properties, comparable to those of metals in some cases. In addition, numerous commercial grades of fiber-reinforced PPA-based composites have been proposed by manufacturers as suitable for the fabrication of products for the automotive and aerospace industries, electronics, etc. due to their superior corrosion resistance and specific strength.

It is known that loading polymers with CFs and GFs enables us to significantly improve the wear resistance of such composites [12,13]. The reason is the fact that reinforcing fibers are characterized by high strength and stiffness, which allows them to effectively bear loads, reducing both the strain and wear of the thermoplastic matrix [14,15,16,17]. In particular, Shin et al. investigated the effect of short GFs (SGFs) with lengths of 400–500 μm and diameters of 6–8 μm on the tribological characteristics of PA66-based composites in linear tribological contacts [18]. They showed that SGFs increase the softening point of PA66 and improve the wear resistance of the composites by reducing the transfer of the polymer material to counterfaces. However, fragments of fractured SGFs can have an irregular micro-abrasive effect on the friction surfaces of such composites, while some protruding ones regularly scratch the counterfaces [19]. In some cases, the addition of lubricants can be a solution to the problem of using composites with high contents of reinforcing fibers in friction units [20,21].

Advantages of loading polymers with CFs as a reinforcing filler (compared to GFs) are their increased strength, as well as both a lower coefficient of friction (CoF) and scratching effect on the counterface surfaces, which slow down the wearing of the friction unit components [22,23,24]. In some recommendations, manufacturers of polymer composites state that their loading with GFs is not suitable for the components of friction units operated under severe conditions. At the same time, the issues of ensuring interfacial adhesion are relevant, which are solved through the sizing of CFs or by modifying their surfaces [25,26,27,28]. Different authors have presented opposing points of view about the role of short CFs (SCFs) on the tribological characteristics of polymer composites, starting from unproven manifestations of the “self-lubricating” effect and ending with statements about improved strength properties due to dispersion hardening and better running-in of the friction surfaces. Many researchers have noted that fragments of fibers on the composite surfaces can cause severe abrasive wear of counterparts during friction under high loads [1,29]. Thus, studying the role of reinforcing fibers (primarily SCFs) is of undoubted scientific and practical relevance.

A large number of papers have already been devoted to the tribological characteristics of fiber-reinforced PA-based composites. For example, Kim et al. studied the effect of loading 10–50 wt.% GFs with lengths of 3–4 mm and diameters of 12–15 μm on the tribological characteristics of PA12-based ones in linear tribological contacts [30]. They showed that both the CoF levels and wear rate (WR) values of the composites decrease by increasing the contents of GFs. The greatest wear resistance was achieved after loading with 30 wt.% GFs. In addition, the effect of the orientation of the GFs on the tribological characteristics of the PA12-based composites was reported. If GFs are oriented perpendicular to the sliding direction, lower WR values are observed. However, it has been noted that the tribological characteristics of the PA12-based composites depend more on both filler content and temperature in the tribological contacts than on the orientation of the CFs (tribological testing conditions: block-on-ring scheme; counterface (ring): medium carbon steel (Ra ≈ 0.2 μm); load: 500 N; velocity: 100 rpm).

In [31], Li et al. studied the effect of loading 10–30% SCFs with a length of 105 µm and a diameter of 7 µm on the tribological characteristics of PA6-based composites. As a result, WR values were reduced, so the greatest wear resistance was achieved at their content of 20%. The authors explained this phenomenon as follows. When the content of CFs has exceeded a certain value, the structures of the PA6-based composites have become heterogeneous. The wear resistance of the composites has been improved since the CFs are more susceptible to cracking and fracturing (tribological testing conditions: ball-on-block scheme; room temperature; counterface (ball): CGr15 steel; load: 6–15 N; reciprocating sliding frequency: 1–12 Hz). In addition, Bijwe et al. investigated the effect of loading PA12 with both SCFs and PTFE on the tribological characteristics of such composites [32]. The WR values were decreased by increasing the content of the CFs. On the other hand, loading with PTFE enables us to additionally improve the tribological characteristics of PA12-based composites. The best results have been obtained after simultaneous loading with 30% CFs and 20% PTFE. In this case, the CoF level and WR value were 0.28 and 0.735 × 10^−6^ mm^3^/N·m, respectively (tribological testing conditions: pin-on-disk scheme; counterface (disk): steel (Ra ≈ 0.5 μm); load: 150 N; velocity: 1 m/s).

The replacement of energy sources, primarily in transport engineering, demands the design of high-strength polymer composites for operating in tribological units. In this regard, fiber-reinforced PPA-based composites may be of undoubted interest, for example, for the manufacturing of sliding bearings for railway vehicles. However, materials for tribological purposes operating under sliding friction conditions, as a rule, possess low-strength properties. Accordingly, the contradictory task of combining virtually opposite requirements arises in designing such composites. In some cases, cages for heavily loaded sliding bearings in railway vehicles (for the manufacture of which PPA can be used) are characterized by insufficient lubrication conditions during their operation. For this reason, it is necessary to qualify their tribological characteristics under both dry-friction and oil-lubricated conditions.

Unlike the strength properties, which are volumetric (and actually controlled by the structure of a bulk material), the tribological characteristics of fiber-reinforced PPA-based composites are the “surface” ones that are affected by several parameters:

- The lengths of reinforcing fibers. They should ensure uniform volumetric and directional distributions. In increasing their length, it is more difficult to avoid agglomeration, which causes anisotropy, primarily in the mechanical properties [33].
- The filler contents. Their enhancement is accompanied by an increase in the strength properties, but heterogeneity of the structure and functional characteristics arises, especially in viscous polymer matrices. Therefore, both numbers and orientations of fibers protruding on the friction surfaces can play decisive roles [33].
- Both the mechanical and rheological properties of the polymer matrix, which should uniformly fill the composite volume due to its low viscosity, including the spaces between fibers. On the other hand, great mechanical loads from counterparts, which are easily supported by fibers, can damage or fracture the polymer “interlayers” between such reinforcing inclusions on the friction surfaces. In this case, it is impossible to prevent their movement under shear stresses transmitted from the sliding counterfaces [34].

The aim of this study was to investigate the tribological characteristics of commercially available high-strength PPA-based composites with great contents (30–50 wt.%) of CFs and GFs in the point and linear tribological contacts against the metal and ceramic counterparts under the dry-friction and oil-lubricated conditions at various *P* loads and *V* sliding speeds. The lengths of CFs and GFs were varied simultaneously with their contents, assuming the possibility of improving the load-bearing capacity (strength) of the composites [35]. Samples were fabricated from granules by injection molding.

According to the authors, the added value of this study is related to establishing wear mechanisms of PPA/SC composites under various loading schemes and conditions. In addition, it might be considered as a practical guide for the application of commercially available fiber-reinforced PPA structural grades for fabricating components of friction units presented in the form of Ashby diagrams.

This article is structured as follows. Section 2 provides a description of materials and research methods. Section 3.1 describes the mechanical properties of the studied composites, while Section 3.2 is devoted to their tribological characteristics in the linear contacts. Section 3.2.1 contains the results obtained in the metal–polymer tribological contact under dry-friction conditions. Then, the composite with the greatest tribological characteristics was tested in more detail. In particular, Section 3.2.2 includes the data after the tests in the metal– and ceramic–polymer tribological contacts under dry-friction and oil-lubricated conditions at different *P* loads, while the variable parameter was the *V* sliding speed in Section 3.2.3. Section 3.3 presents the tribological characteristics in the point tribological contact. All obtained results are discussed in Section 4, preceding some drawn conclusions in Section 5.

## 2. Materials and Methods

Samples of neat PPA and PPA-based composites were fabricated from granules of different grades (Table 2) by injection molding using an “RR/TSMP” laboratory machine (RAY-RAN TEST EQUIPMENT LTD, Warwickshire, UK) at a cylinder temperature of 360 °C, a mold temperature of 150 °C, and a pressure of 9 bar.

It should be noticed that short (both GF and CF) fibers were loaded into granules as a separate filler. Thus, their length did not change during the subsequent granulation. At the same time, PPA composites with continuous fiber were pultruded first with further processing (granulation) into granules. In doing so, the fibers’ lengths were comparable to the length of the granules. No additional surface treatment was applied to the fibers.

Since initial blanks of PPA composites were injection-molded with further cutting of test samples from the plates using a CNC milling machine (Purelogic R&D LLC, Voronezh, Russia), no preferential fiber orientation was realized.

The densities of the samples were assessed by hydrostatic weighing, that is, by comparing masses of equal volumes of the composites and distilled water. For such products, the method provided a measurement accuracy of about 0.1%.

The tensile strength properties were measured with an “Instron 5582” electromechanical testing machine according to ASTM D638 [36]. Dog-bone samples that were 63.5 ± 0.4 mm long, 9.5 ± 3.2 mm wide, and 3.2 ± 0.4 mm thick were tested. Their gauge lengths were 9.6 ± 0.5 mm long, 3.2 ± 0.4 mm wide, and 3.2 ± 0.5 mm thick, while the extensometer base was 7.6 ± 0.3 mm, the distance between the clamps was 25.4 ± 5 mm, and the curvature radius was 12.7 ± 1 mm. The flexural strength values were determined in three-point bending tests using the “Instron 5582” electromechanical machine in accordance with ASTM D790 [37] (the dimensions of the tested specimens were 80 × 10 × 4 mm). For the sake of statistics, at least five samples of each type were tested.

The shore *D* hardness was determined using an “Instron 902” setup according to ASTM D2240 [38].

The Charpy impact strength was measured using a “KM-5” pendulum impact tester (ZIP LLC, Ivanovo, Russia). The dimensions of the specimens were 80 × 10 × 4 mm according to the Russian state standard GOST 4647-80 [39]. The number of specimens of each type was four. Then, the average value was calculated by statistically processing the obtained data.

The required surface quality of the specimens for tribological testing was achieved by grinding with sandpapers up to P2000 (ISO 6344) [40]. 

In the point contact (the “ball-on-disk” scheme) under the dry-friction conditions, tribological tests were conducted using a “CSEM CH-2000” tribometer (CSEM, Neuchatel, Switzerland) in accordance with ASTM G99 [41] at a *P* load of 10 N and a *V* sliding speed of 0.3 m/s. The diameter of both GCr15 bearing steel and Al_2_O_3_ ceramic counterparts was 6 mm. The test distance was 0.3 km, and the rotation path radius was 10 mm, so the circular speed was 286 rpm. The roughness of GCr15 bearing steel and Al_2_O_3_ ceramic balls was Ra = 0.02 μm, which were evaluated with a “New View 6200” profilometer (Zygo, Middlefield, CT, USA).

In the linear contact (the “block-on-ring” scheme) under the dry-friction conditions, the tribological characteristics were assessed using a “2070 SMT-1” friction testing machine (Tochpribor Production Association, Ivanovo, Russia) according to ASTM G77-17 [42]. The *P* loads were 60, 120, and 180 N, while the *V* sliding speed was 0.3 m/s. The test distance was 1 km. Counterfaces with a diameter of 35 mm were made of GCr15 bearing steel and the Al_2_O_3_ ceramics with the surface roughness of Ra = 0.2–0.25 μm. The block’s dimensions were as follows: length 16 mm × width 6.4 mm × height 10 mm. WR values were determined by measuring the wear track volume using a contact (stylus) “Alpha-Step IQ” surface profiler (KLA-Tencor, Milpitas, CA, USA). During the tribological studies, at least four samples of each type were tested.

The average value of the thermal conductivity of the GCr15 steel is ~120 W/mK, while it is equal to ~25 W/mK for ceramic Al_2_O_3_.

**Figure 2:**
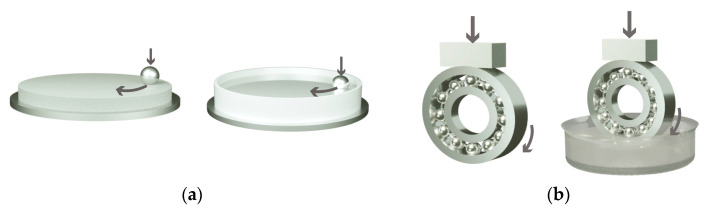
Schematics of (**a**) “ball-on-disk” and (**b**) ”block-on-ring” under dry-friction and oil-lubricated conditions.

”MDPN-S” electrical insulating synthetic oil (the “FOXY” brand, Necton Sea LLC, Moscow, Russia) was used as a lubricant. The kinematic viscosity of the oil was 18–27 mm^2^/s (at *T* = 50 °C), while the density was 850 kg/mm^3^ (at *T* = 20 °C). In order to implement oil-lubricated testing conditions, the lower part of the counterfaces was put in a plastic tank with the oil (Figure 2).

The counterpart temperature was assessed using a “CEM DT-820” non-contact infrared (IR) thermometer (Shenzhen Everbest Machinery Industry Co., Ltd., Shenzhen, China). The diameter of a region under the measurement was 15 mm.

After the tribological tests, the wear track surfaces were examined with a “Neophot 2” optical microscope (Carl Zeiss, Jena, Germany) equipped with a “Canon EOS 550D” digital camera (Canon Inc., Tokyo, Japan).

The topography of the wear tracks was observed using a “LEO EVO 50” scanning electron microscope (SEM; Carl Zeiss, Germany) at accelerating voltages of 10, 20, and 30 kV.

The structures of the samples were investigated on their cleavage surfaces (mechanically fractured in liquid nitrogen after notching) with “LEO EVO 50” SEM at an accelerating voltage of 20 kV.

The water absorbency of the composites was determined in accordance with ISO 62:2008 [43] and the Russian state standard GOST 4650-2008 [44].

## 3. Results

### 3.1. Mechanical Properties

**Figure 3:**
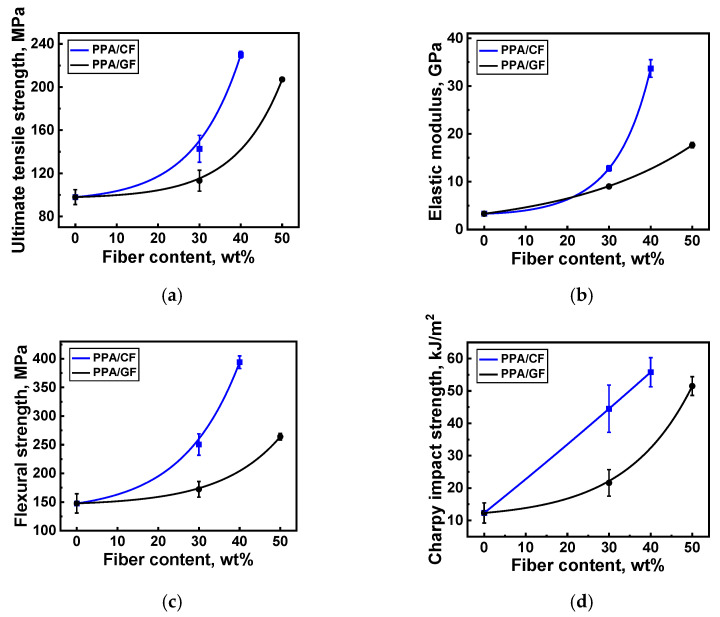
The dependences of the mechanical properties of neat PPA and the PPA-based composites on the fiber contents: (**a**) ultimate tensile strength, (**b**) elastic modulus, (**c**) flexural strength, and (**d**) Charpy impact strength.

Table 3 presents the physical and mechanical properties of neat PPA and the PPA-based composites. In addition, Figure 3 shows their dependences on the fiber contents. It could be concluded that even neat PPA possessed rather great characteristics since its ultimate tensile strength and elastic modulus were 97.7 ± 6.9 MPa and 3.3 ± 0.1 GPa, respectively. After loading PPA with 30 wt.% SCFs at an aspect ratio (AR) of 200, the ultimate tensile strength and the elastic modulus increased up to 142.7 ± 12.5 MPa and 12.9 ± 0.6 GPa, respectively. Loading PPA with 30 wt.% SGFs (AR = 200) gave a more negligible effect; in particular, the ultimate tensile strength was 113.1 ± 9.7 MPa, while the elastic modulus was 9.0 ± 0.3 GPa. According to the authors, the reason was the higher density of SGFs of 2.5 g/mm^3^, compared to that of 1.75 g/mm^3^ for SCFs, the volume fraction of which at the same weight was less than 17.4% in contrast to 24.8% for SCFs.

In the composites with the higher contents of reinforcing fibers, their lengths were greater as well: the AR was 1000 for CCFs at the content of 40 wt.% (30 vol.%), while it was 445 for CGFs at the content of 50 wt.% (31.6 vol.%). The ultimate tensile strength and the elastic modulus were 240 ± 3 MPa and 33.7 ± 1.9 GPa, respectively, for the PPA/40CCF composite, while they were 207 ± 1 MPa and 17.7 ± 0.6 GPa for the PPA/50CGF one.

**Figure 4:**
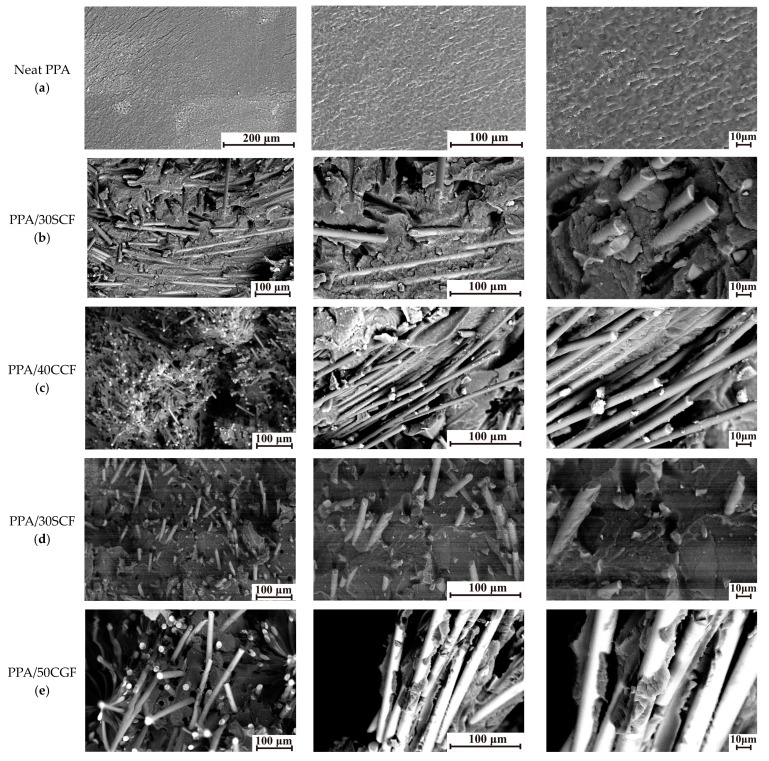
SEM micrographs of structures of neat PPA and PPA-based composites. (**a**) Neat PPA, (**b**) PPA/30SCF, (**c**) PPA/40CCF, (**d**) PPA/30SCF, (**e**) PPA/50CGF.

Based on an analysis of these data, two general trends were identified: at equal weight contents, the addition of SCFs provided a greater contribution to the increase in the ultimate tensile strength, compared with SGFs. Enhancing the aspect ratios of both CFs and GFs by several times was also accompanied by its improvement. Similar trends were typical for the flexural strength and Charpy impact strength (Figure 3c,d). However, as shown in Figure 4, increases in both the lengths and contents of reinforcing fibers led to significant heterogeneity of the composite structures with obvious anisotropy of the fiber orientations. According to Figure 4, great adhesion between reinforcing fibers and the polymer matrix was observed in all studied cases. This fact was associated with the excellent manufacturability of PPA due to its high fluidity and the presence of a sizing agent on the fiber surfaces. Since all PPA-based composites were fabricated via the same production route, reinforcing fibers were predominantly oriented in the casting plane.

After loading PPA with 30 wt.% SCFs (AR = 200), some areas with their predominant orientation were observed (Figure 4b). In the PPA/40CCF composite (AR = 1000), reinforcing fibers were characterized by a predominant orientation (laying) and their distribution was uneven, according to Figure 4c. At 30 wt.% SGFs (AR = 200), the distribution of reinforcing fibers was uniform and directional (Figure 4d). The reason could be the greater fragility of SGFs, which resulted in their fracture upon manufacturing the PPA/30SGF composite. In the PPA/50CGF one (AR = 445), the distribution of reinforcing fibers (Figure 4e) was generally similar to that in the PPA/40CCF composite (Figure 4c), since both contents and lengths of these fillers were similar. As shown below, the identified distribution pattern of reinforcing fibers, primarily in the surface layers of wear tracks, also determined WR values.

### 3.2. Tribological Characteristics in Linear Contacts

#### 3.2.1. The Metal-Polymer Contacts under the Dry Friction Conditions

**Figure 5:**
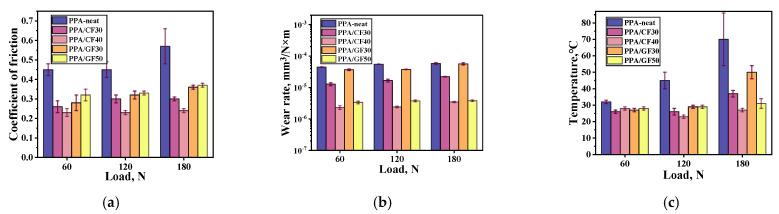
Bar graphs of the tribological characteristics of neat PPA and the PPA-based composites at different *P* loads: (**a**) CoF levels, (**b**) WR values, (**c**) counterface temperatures; the steel counterface, the “block-on-ring” scheme, the dry sliding friction.

Since a promising direction for using the studied composites was their implementation as sliding bearing components, trial tribological tests were conducted in available linear contacts according to the “block-on-ring” scheme. Initially, the dry friction conditions were applied when testing against the metal counterface at the constant *V* sliding speed of 0.3 m/s but the varied *P* loads of 60, 120 and 180 N. The results of the tribological tests are summarized in Table 4 and Figure 5. According to these data, the PPA/40CCF composite was characterized by the highest WR value of 2.33 × 10^−6^ mm^3^/N·m and the minimum CoF level of 0.23 at *P* = 60 N. After the threefold increase in the *P* load, the CoF level remained virtually constant, while the WR value increased by 1.5 times up to 3.47 × 10^−6^ mm^3^/N·m. For the PPA/50CGF composite, CoF levels of 0.32–0.37 were higher by 1.5 times at stable WR values of (3.36–3.81) × 10^−6^ mm^3^/N·m. With the lower both contents and lengths of reinforcing fibers, the composites had significantly lower tribological characteristics. In addition, it should be pointed out that the spread of COF and WR values are of importance when repeatability and stability of tribological testing are concerned. Thus, higher spread of experimental values indicates a more inhomogeneous mechanism and lower repeatability. This means that, when two materials have a similar COF, the one with the lower spread would be preferred for an application. However, their discussion has not been performed in the paper for the sake of saving the space. 

**Figure 6:**
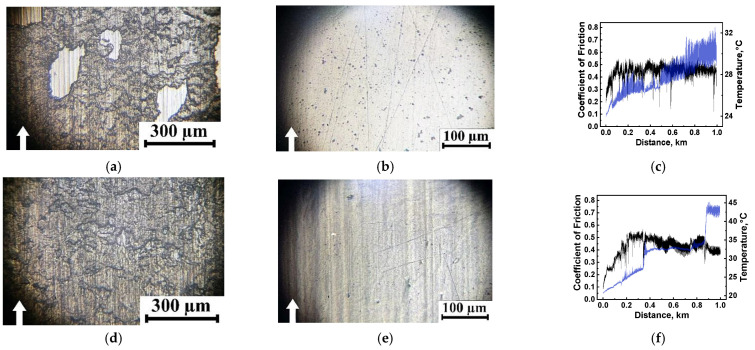

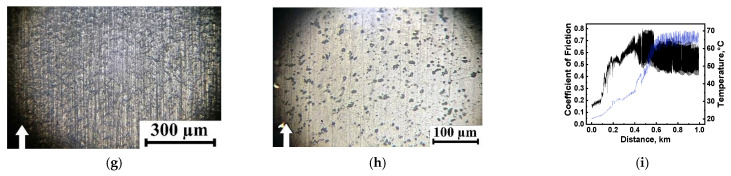
The optical micrographs of the surfaces on the steel counterface (**b**,**e**,**h**) and on the wear tacks on neat PPA (**a**,**d**,**g**), as well as the CoF (black curve) and temperature (blue curve) versus distance dependences (**c**,**f**,**i**); the “block-on-ring” scheme, the dry sliding friction; white arrows show the sliding direction.

Figure 6 shows optical micrographs of the surfaces on the steel counterpart and the wear tacks on neat PPA, as well as the dependences of the CoF levels and the counterface temperatures on the test distance. In all cases, a transfer layer with varying degrees of continuity formed on the steel counterpart surface (Figure 6a,d,g). This fact reflected great damage to the surfaces on neat PPA (Figure 6b,e,h), the level of which increased in proportion to the applied *P* load. Such a trend was significant as it indicated the relative ease of separation of the polymer from the friction surface and subsequent consolidation into a continuous transfer film. However, it was not possible to adhere it on the steel counterpart surface for a sufficiently long time, as evidenced by periodic “drops” and fluctuations of the CoF levels (Figure 6c,e,i), which did not correlate with the changing kinetics (growth) in the steel counterpart temperature. When it was heated, fluctuations and abrupt changes in the CoF levels were characteristic, especially at the high loads (Figure 6f,i). For a given, rather inertially changing parameter, this phenomenon was atypical and indicated the non-stationary nature of processes developing in the tribological contacts.

**Figure 7:**
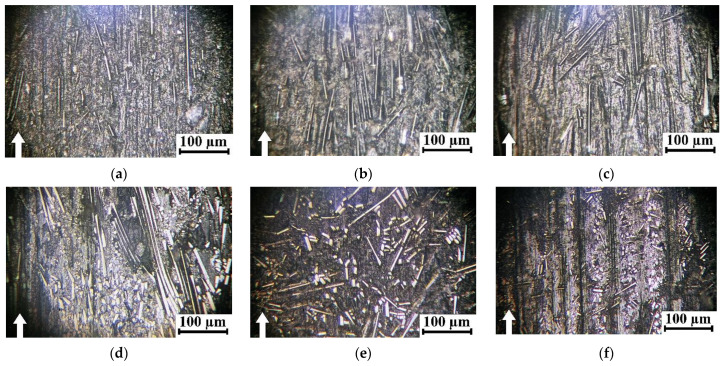

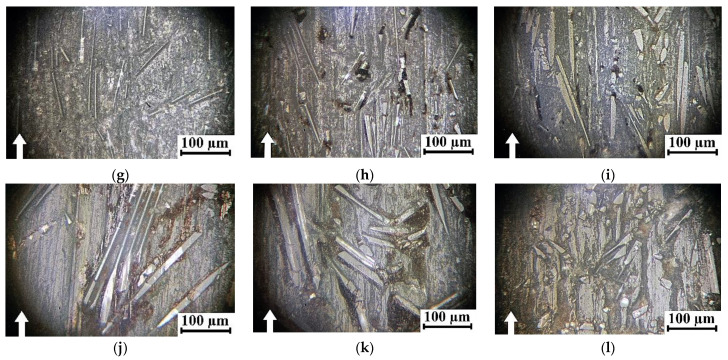
The optical micrographs of the wear tracks on the PPA/30SCF (**a**–**c**), PPA/40CCF (**d**–**f**), PPA/30SGF (**g**–**i**) and PPA/50CGF (**j**–**l**) composites after the tribological tests at *P* = 60 (**a**,**d**,**g**,**j**), 120 (**b**,**e**,**h**,**k**) and 180 (**c**,**f**,**i**,**l**) N, *V* = 0.3 m/s; the steel counterface, the “block-on-ring” scheme, the dry sliding friction.

**Figure 8:**
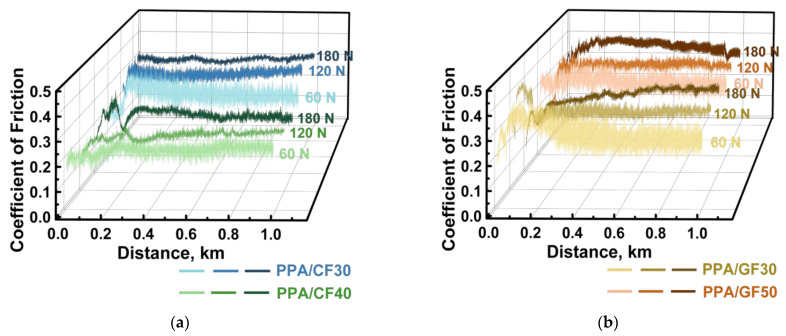
The CoF versus distance dependences for the PPA/30SCF and PPA/40CCF (**a**) as well as PPA/30SGF and PPA/50CGF (**b**) composites at the *P* loads of 60, 120 and 180 N; the steel counterface, the “block-on-ring” scheme, the dry sliding friction.

Figure 7 shows optical micrographs of the wear tracks on the studied composites after the tribological tests, while their CoF versus distance dependences are presented in Figure 8. In all cases, CoF levels were constants at the steady state wearing stage (see also Table 4). After testing the PPA/30SCF composite, reinforcing fibers visually maintained their integrity, virtually at all applied loads (Figure 7a–c), while they were partially fractured in the PPA/40CCF one (Figure 7d–f). This fact could increase WR values due to reinforcement, according to the idea about tribological layers suggested by the authors in [45]. Nevertheless, the process of mixing fragments of fractured fibers and the polymer matrix was difficult with such both the high content of SCFs and the low proportion of PPA. For this reason, only fractured SCFs were found using the optical microscopy. In the “Discussion” section, this phenomenon was analyzed via the SEM examinations. After loading PPA with 30 wt.% SGFs (AR = 200), reinforcing fibers remained intact after testing at all applied *P* loads (Figure 7g–i). In addition, low damage to the PPA/50CFG composite was observed (Figure 7j–l), which was accompanied by grinding of reinforcing fibers due to their greater thickness and, possibly, better retention within the polymer matrix.

**Figure 9:**
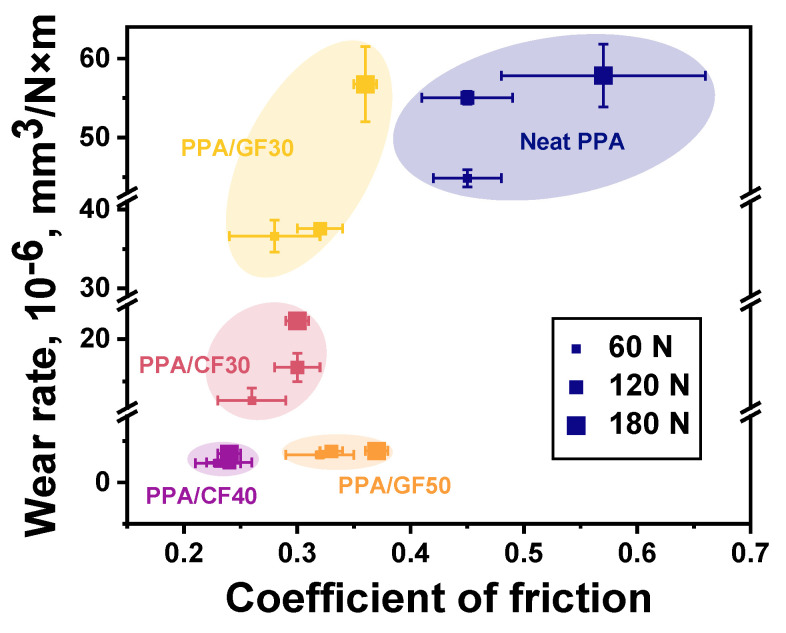
The WR versus CoF Ashby diagram; the steel counterface, the “block-on-ring” scheme, the dry sliding friction. Colored bubbles cluster composites of the same content.

According to Figure 8, the changing kinetics of the CoF levels were not fundamentally different after increasing the *P* load from 60 N up to 120 and 180 N. For these cases, both CoF levels and WR values are summarized in Figure 9 as an Ashby diagram [46]. Based on an analysis of the obtained data, the PPA/40CCF composite was characterized by the most advanced tribological characteristics. In particular, the WR value was similar to that for the PPA/50CGF one at the noticeably lower CoF level. So, the only PPA/40CCF composite was analyzed in detail under different tribological conditions then.

#### 3.2.2. The Metal- and Ceramic-Polymer Contacts under the Dry Friction and Oil Lubricated Conditions

**Figure 10:**
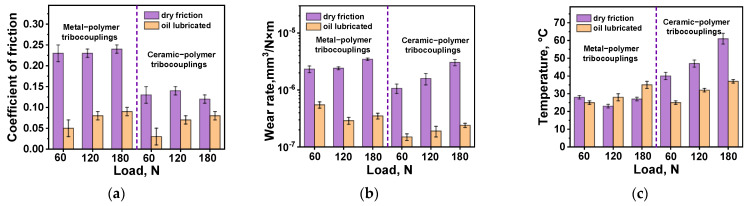

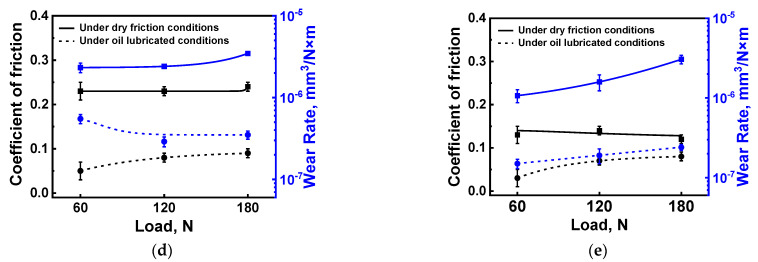
The summarized data on the tribological characteristics of the PPA/40CCF composite at the applied *P* loads: the bar diagrams (**a**–**c**) as well as the CoF (black curves) and WR (blue curves) versus load dependences (**d**,**e**); the steel (**d**) and ceramic (**e**) counterfaces.

Both steel and ceramic counterfaces were used in the tribological tests for comparison. The steel one was characterized by a lower hardness but a greater chemical reactivity to PPA. The ceramic counterface was less exposed to the mechanical effects of the composite and interaction with it during tribological oxidation of the polymer (due to frictional heating). In addition, wear resistance of the PPA/40CCF composite was analyzed under the oil lubricated conditions, which simulated its application in railway bearings. The determined tribological characteristics are presented in Table 5. In addition, these data are shown graphically as bar diagrams (Figure 10a–c) as well as dependences of the CoF levels and the WR values on the applied *P* loads (Figure 10d,e, respectively).

According to Figure 10a, the CoF levels were almost two times lower upon testing against the ceramic counterface compared to those for the steel one under the dry friction conditions. Increasing the *P* load was not accompanied by a noticeable change in the CoF levels for the steel counterface (Figure 10b,d), while the WR values enhanced almost linearly for the ceramic one (Figure 10b,e). This fact could be caused by rising the counterpart temperature due to its low thermal conductivity. Note that the implemented non-contact method to estimate the counterface temperature was integral, so heating could exceed the glass transition level of PPA in the contact spots, intensifying strains of the polymer matrix.

**Figure 11:**
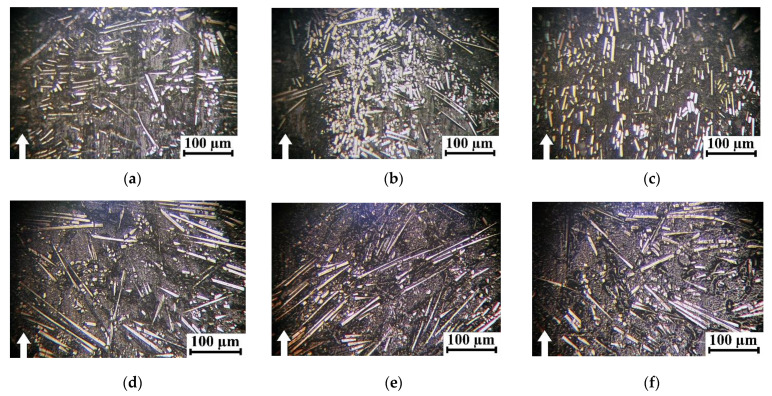

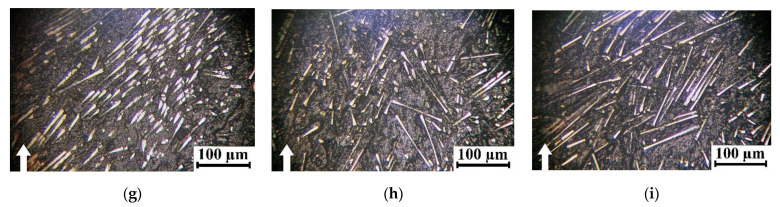
The optical micrographs of the wear track surfaces on the PPA/40CCF composite after the tribological tests against the ceramic counterface under the dry friction conditions (**a**–**c**), as well as after the test against the steel (**d**–**f**) and ceramic (**g**–**i**) counterfaces under the oil lubricated conditions; the linear contact, *P* = 60 (**a**,**d**,**g**), 120 (**b**,**e**,**h**) and 180 (**c**,**f**,**i**) N, *V* = 0.3 m/s. White arrows show the sliding direction.

**Figure 12:**
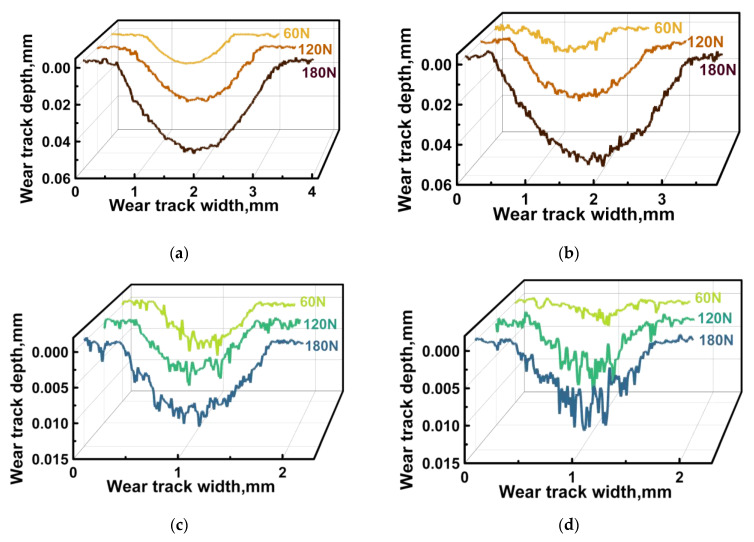
The wear track profiles on the PPA/40CCF composite after the tribological tests against the steel (**a**,**c**) and ceramic (**b**,**d**) counterfaces under the dry friction and oil lubricated conditions; the linear tribological contact, *P* = 60, 120 and 180 N, *V* = 0.3 m/s.

Under the oil lubricated conditions, the WR values and the CoF levels (comparable under identical loads for both counterfaces) were expectedly lower (Figure 10a,d). At the same time, the WR values reduced upon testing against the ceramic counterface (Figure 10b,e). This fact was not associated with the influence of temperature, since although it rose with increasing the *P* load, it was not particularly different for the steel and ceramic counterfaces (Figure 10c). Possible reasons for this phenomenon are discussed below, when analyzing both the surface topography of the wear tracks and their profiles (Figure 11 and Figure 12, respectively).

**Figure 13:**
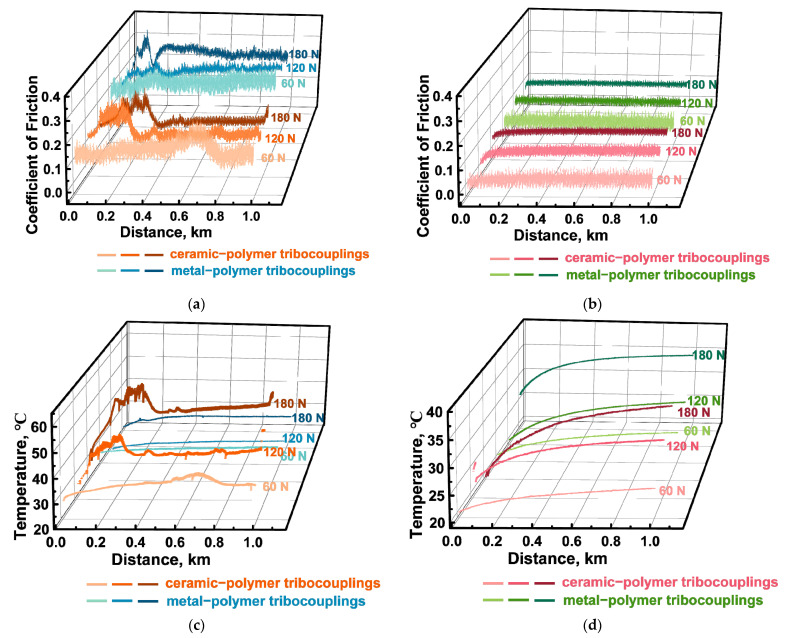
The CoF (**a**,**b**) and temperature (**c**,**d**) versus distance dependences for the PPA/40CCF composite during the tribological tests against the steel and ceramic counterfaces under the dry friction (**a**,**c**) and oil lubricated (**b**,**d**) conditions; the linear tribological contact, *P* = 60, 120 and 180 N, *V* = 0.3 m/s.

Under the dry friction conditions against the ceramic counterface, CoF levels were constant despite the varied *P* loads. Moreover, they were lower than those of ~0.12–0.14 for the steel counterface. Since the only difference was the material (inert ceramics), the authors attributed this phenomenon to its negligible chemical interaction. At the same time, the changing kinetics in the CoF levels were not stable at all applied *P* loads (Figure 13a). On the wear track surfaces, fragmented (fractured) CCFs were observed in all cases (Figure 11a–c), the distribution of which was not always uniform. It should be noted that a characteristic feature of the wear tracks was the local adherence of debris of the polymer matrix (Figure 11a–c), which was confirmed by comparing the wear track profiles shown in Figure 12a,b.

Under the oil lubricated conditions, WR values decreased significantly and were comparable for both counterparts (Figure 10d,e). In these cases, SCFs protruding above the wear track surfaces generally retained their integrity after the tribological tests against both steel (Figure 11d–f) and ceramic (Figure 11g–i) counterfaces at all applied *P* loads. For the steel counterface, a certain trend toward a decrease in this effect was characteristic as the *P* load enhanced, while its increase was obvious in the second case. In fact, “grinding” of SCFs occurred, as evidenced by both different diameters of such reinforcing fibers and their varied reflectivity (Figure 11d–i).

After the tribological tests against the steel and ceramic counterfaces, wear track profiles were also informative (Figure 12c,d, respectively). In particular, the “toothed” profile (characterized by an increase in the degree/height of steps as the *P* load enhanced) indicated that the presence of oil could be accompanied by “erosion” of the polymer matrix, while SCFs continued to bear the *P* loads transmitted from the counterfaces. Upon testing, CoF levels were consistently low for both counterfaces (Figure 13), which was obvious from the point of view of friction under the oil lubricated conditions.

**Figure 14:**
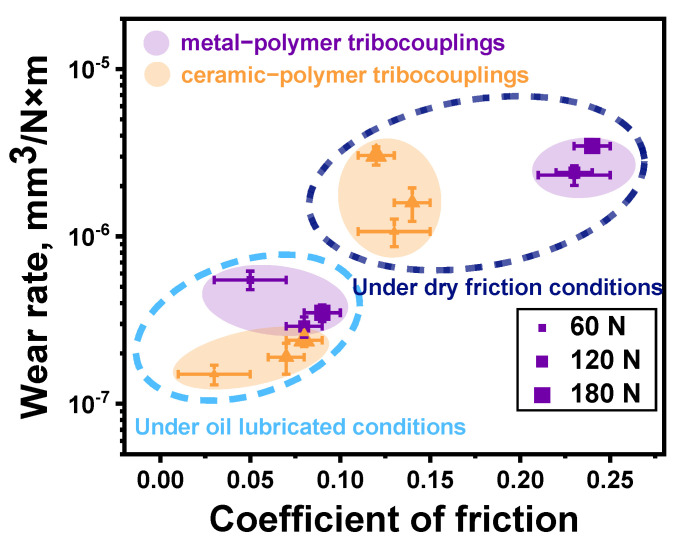
The CoF versus WR Ashby diagram for the PPA/40CCF composite under the dry friction and oil lubricated conditions; the steel and ceramic counterfaces, the linear tribological contact, *P* = 60, 120 and 180 N, *V* = 0.3 m/s. Colored bubbles cluster composites of the same content.

The above-described data are summarized in Figure 14 as an Ashby diagram. Expectedly, the greater tribological characteristics were obtained under the oil lubricated conditions, but varying the *P* load was not accompanied by a significant change in both CoF levels and WR values upon testing. Since the WR value could be affected by the *V* sliding speed in addition to the *P* load (rising the counterface temperature), this assumption was verified by additional tribological tests.

#### 3.2.3. The Linear Tribological Contact at Different Sliding Speeds

**Figure 15:**
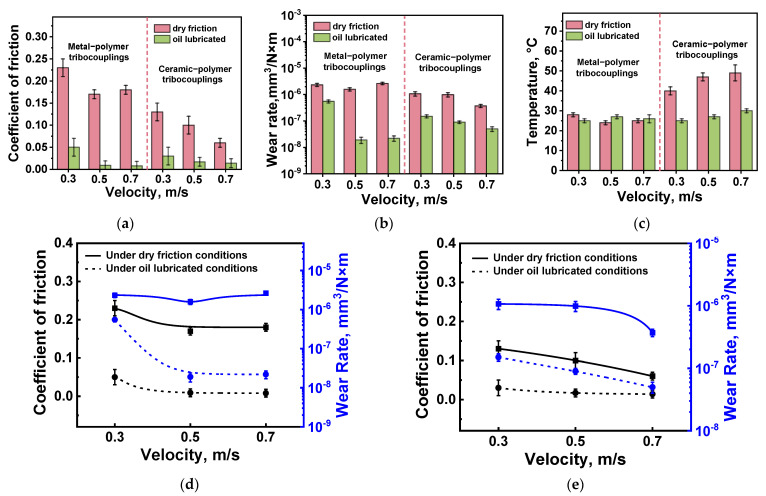
The summarized data on the tribological characteristics of the PPA/40CCF composite at different *V* sliding speeds: the bar diagrams (**a**–**c**) as well as the CoF (black curves) and WR (blue curves) versus sliding speed dependences (**d**,**e**); the steel (**d**) and ceramic (**e**) counterfaces.

The determined tribological characteristics are presented in Table 6. In addition, they are shown as diagrams in Figure 15a–c and dependences of the CoF levels and the WR values on the *V* sliding speed in Figure 15d,e. According to Figure 15a, the CoF levels decreased with increasing the *V* sliding speed upon the tribological tests under the dry friction conditions against both counterfaces. Moreover, they were several times lower for the ceramic one (Figure 15a,d,e). On the contrary, the WR values practically did not change as the *V* sliding speed enhanced (Figure 15b), but it was slightly higher for the steel counterfacet than that for the ceramic one. Unlike for the tests at various *P* loads, these results could not be explained by the influence of the counterface temperatures, since the WR values were almost constant over the entire range of the applied *V* sliding speeds upon the tribological tests against the steel counterpart, while they increased almost linearly in the second case (Figure 15c).

Under the oil lubricated conditions, both CoF levels and WR values decreased. The CoF levels of >0.05 were low and comparable for both counterfaces, decreasing slightly as the *V* sliding speed increased (Figure 15d,e). An unusual result was obtained at higher *V* sliding speeds, when the WR value was lower for the steel counterface than that for the ceramic one (Figure 15b,d,e). It should be noted that they were approximately the same throughout the studied range from 0.3 up to 0.7 m/s upon the tribological tests against the ceramic counterface (Figure 15b). The presence of oil neutralized rising the counterpart temperatures for both materials at all the applied *V* sliding speeds (Figure 15c).

**Figure 16:**
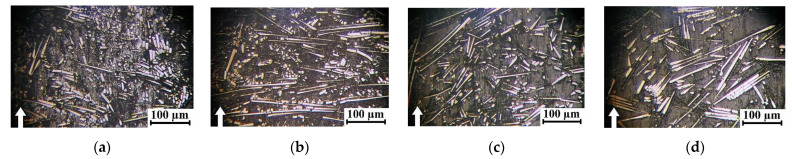

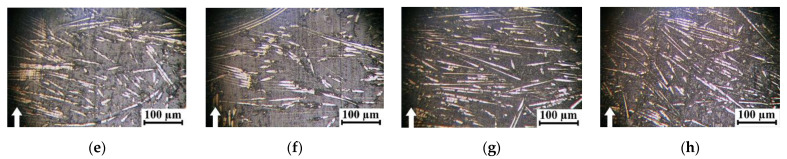
The optical micrographs of the wear track surfaces on the PPA/40CCF composite after the tribological tests against the steel (**a**,**b**,**e**,**f**) and ceramic (**c**,**d**,**g**,**h**) counterfaces; the dry friction (**a**–**d**) and oil lubricated (**e**–**h**) conditions; the linear tribological contact, *P* = 60 N, *V* = 0.5 (**a**,**c**,**e**,**g**) and 0.7 (**b**,**d**,**f**,**h**) m/s.

**Figure 17:**
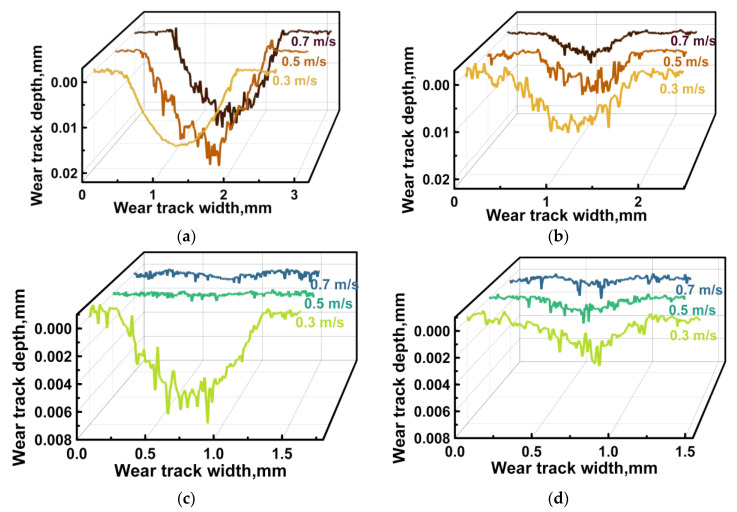
Wear track profiles on the PPA/40CCF composite after the tribological tests against the steel (**a**,**c**) and ceramic (**b**,**d**) counterfaces under the dry friction (**a**,**b**) and oil lubricated (**c**,**d**) conditions; the linear tribological contact, *P* = 60 N, *V* = 0.3, 0.5 and 0.7 m/s.

After the tribological tests under the dry friction conditions, complete or partial fracture of CCFs was observed at WR values of ≥1 × 10^−6^ mm^3^/N·m. They protruded onto the wear track surfaces upon testing the PPA/40CCF composite against both steel (Figure 16a,b) and ceramic (Figure 16c) counterfaces. In these cases, the polymer matrix was either locally eroded or adhered as debris on the wear track surfaces (Figure 16a). This fact was also evidenced by the wear track profiles (Figure 17a,b). Only at the low WR values, CCFs were characterized by “polished” surfaces (Figure 16d and Figure 17a).

**Figure 18:**
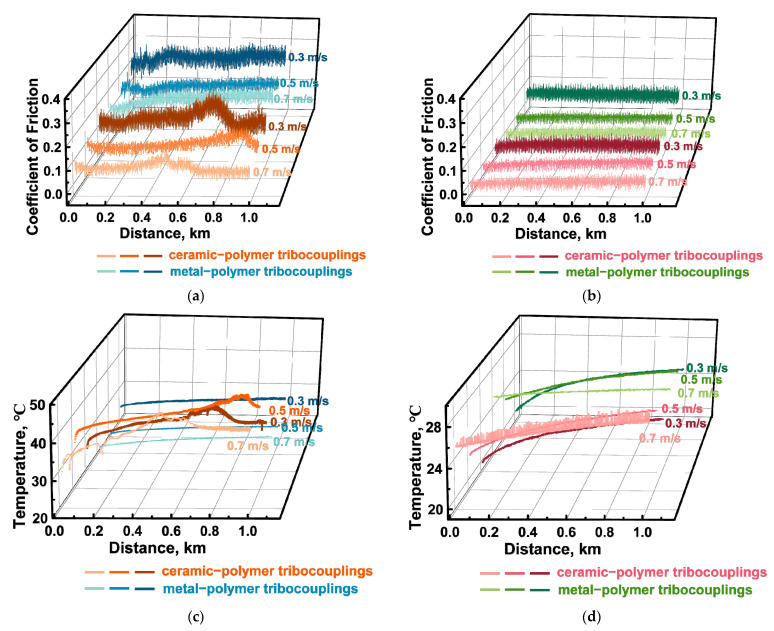
The CoF (**a**,**b**) and temperature (**c**,**d**) versus distance dependences for the PPA/40CCF composite during the tribological tests against the steel and ceramic counterfaces under the dry-friction (**a**,**c**) and oil-lubricated (**b**,**d**) conditions; the linear tribological contact, *P* = 60 N, *V* = 0.3, 0.5 and 0.7 m/s.

CoF levels of ~0.1 were less stable during the tribological tests against the ceramic counterface (Figure 18a). Such a trend was quite consistent with its multiple higher temperatures (Figure 18c) compared to those for the more heat-dissipating steel (Figure 18d).

**Figure 19:**
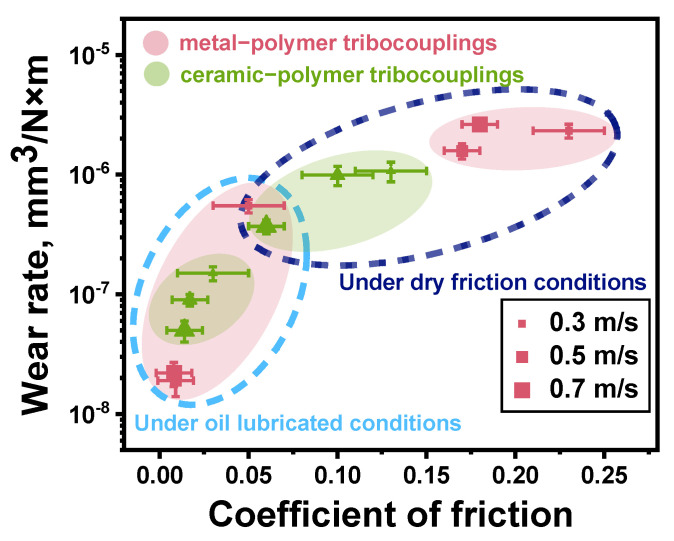
The CoF versus WR Ashby diagram for the PPA/40CCF composite under the dry-friction and oil-lubricated conditions; the steel and ceramic counterfaces, the linear tribological contact, *P* = 60 N, *V* = 0.3, 0.5 and 0.7 m/s. Colored bubbles cluster the same tribological conditions.

The above-described data are summarized in Figure 19 as an Ashby diagram. After varying the *V* sliding speed, several interesting facts should be highlighted. For the tribological tests against the ceramic counterface under the dry-friction conditions, higher tribological characteristics were obtained, but the scatters of both WR values and CoF levels were greater than those for the steel one. Under the oil-lubricated conditions, the best tribological characteristics were registered for the steel counterface at the *V* sliding speeds of 0.5 and 0.7 m/s. At *V* = 0.3 m/s, WR values increased by almost an order of magnitude and were higher than those for the ceramic counterpart. In this tribological contact, they changed in a noticeably smaller range upon testing at all applied *V* sliding speeds. Thus, the changes in both *P* load and *V* sliding speed had a somewhat different effect on the tribological characteristics of the PPA/40CCF composite.

### 3.3. Tribological Characteristics in Point Tribological Contact

**Figure 20:**
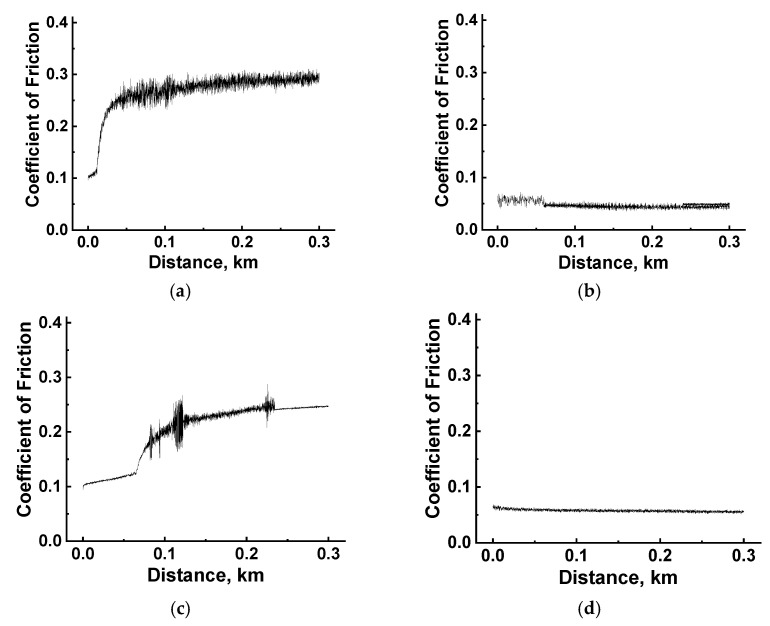
The CoF versus distance dependences for the PPA/40CCF composite upon testing against the steel (**a**,**b**) and ceramic (**c**,**d**) counterfaces under dry-friction (**a**,**c**) and oil-lubricated (**b**,**d**) conditions; the point tribological contact.

As it was reported in a recent paper by the authors [20], HPP-based composites loaded with SCFs at contents above 10% exerted a micro-abrasive effect on counterfaces, leading to either “scratching” or wearing. For the “ball-on-disk” scheme, this phenomenon predominantly manifested itself at both high *P* loads (often exceeding the yield strength of the polymer matrix) and a low *Ra* roughness of ~0.02 µm on the steel counterface surfaces. According to [47], such an issue could be solved by forming a transfer film on their surface. Since this study was devoted, among other things, to the verification of the tribological characteristics of the industrially produced composites, it was of interest to test them in the point tribological contacts. For the PPA/40CCF one, the obtained results are presented in Table 7 and Figure 20. In general, they were both expected and different from those in the linear contacts. Under the dry-friction conditions, CoF levels of 0.24–0.27 were close for both the steel and ceramic counterfaces, while WR values of (5.4–18.9) × 10^−6^ mm^3^/N·m differed by more than three times. Under the oil-lubricated conditions, CoF levels of <0.06 were extremely low for both counterparts. However, an WR value of 0.3 × 10^−6^ mm^3^/N·m for the ceramic counterpart was five times higher than that of 1.6 × 10^−6^ mm^3^/N·m for the steel one. It should be noted that such a negligible WR value was not crucial.

**Figure 21:**
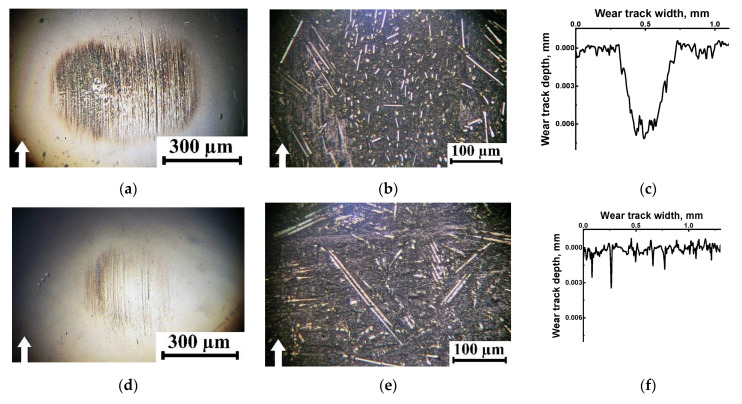

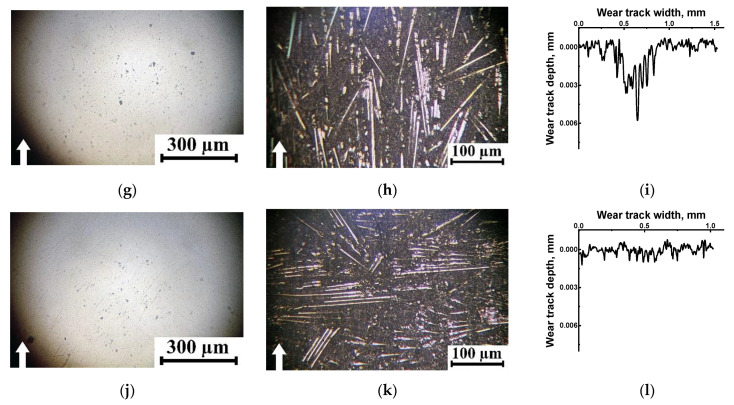
Optical micrographs of the surfaces on the steel counterface (**a**,**d**,**g**,**j**) and on the wear tracks of the PPA/40CCF composite (**b**,**e**,**h**,**k**), as well as their profiles (**c**,**f**,**i**,**l**) after the tribological tests against the steel (**a**–**f**) and ceramic (**g**–**l**) counterfaces under the dry-friction (**a**–**c**,**g**–**i**) and oil-lubricated (**d**–**f**,**j**–**l**) conditions; the point tribological contact.

Under the dry-friction conditions, the changing kinetics of in the CoF levels were very informative. For the steel counterface, they almost immediately reached a constant high value of ~0.27 (Figure 20a). So, it could be considered that the obtained WR value was caused by stable wearing at the steady-state stage. Similar to the linear tribological contact, fragmented (fractured) CCFs were evident on the wear track surface (Figure 21b). This fact corresponds to the formation of a brown-black colored film on the steel counterpart surface, most likely from debris of tribologically oxidized PPA, which allowed them to be adhered (Figure 21a).

It should be noted that only a few shallow scratches were observed on the steel counterface surface after the tribological test under the dry-friction conditions. This result was unexpected, since the high WR value was caused by the protrusion of fragmented CCFs above the wear track surface under such tribological conditions. Accordingly, their expected effect had to be the intensified wear of the steel counterface. However, such a phenomenon was not observed. For the ceramic counterface, the first third of the test distance was characterized by a low CoF level of ~0.1 (Figure 20c), gradually increasing and accompanied by fluctuation then. Nevertheless, this phenomenon did not lead to an increase in the WR value, and the ceramic counterpart surface remained (visually) unchanged (Figure 21g).

After the tribological tests against both counterfaces under the oil-lubricated conditions, no damage to the PPA/40CCF composite was found (Figure 21e,f). At the same time, weak signs of interaction (micro-scratches) of CCFs with the steel counterface were evident on its surface (Figure 21d). On the wear track surface, several polished CCFs were present, while their integrity was maintained as expected (Figure 21e). In general, a similar pattern was characteristic for the tribological test against the ceramic counterface under the oil-lubricated conditions. In particular, any signs of wear were difficult to detect as well (Figure 21k,l), so the authors did not discuss quantitative agreement/mismatch compared to the results obtained for the steel counterface.

Based on the above, the PPA/40CCF composite was characterized by poor wear resistance in the point tribological contact under the dry-friction conditions, which was improved by adding the oil lubricant. In this case, only shallow (micro)scratches were observed of the counterpart surface, which did not lead to an increase in the WR values.

## 4. Discussion

The key aim of this study was to test the highly loaded (up to 40–50 wt.%) polymer composites with the matrix possessing both improved strength properties and great MFI (at the relatively high glass transition temperature of ~120 °C). In addition, the PPA/40CCF composite was classified by the manufacturer as filled with continuous fibers (with a finite length of about 8 mm).

**Figure 22:**
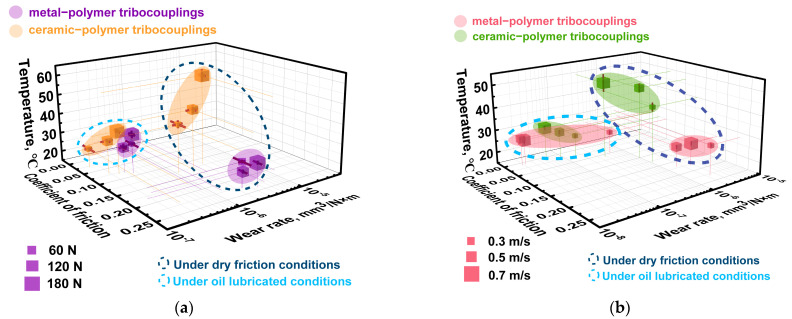
Three-dimensional Ashby diagrams summarizing the relationships between the CoF levels, the WR values, and the counterpart temperatures for the PPA/40CCF composite at the applied both *P* loads (**a**) and *V* sliding speeds (**b**).

In order to determine the relationships between the CoF levels, the WR values, and the counterpart temperatures for the PPA/40CCF composite at the applied both *P* loads and *V* sliding speeds, 3D Ashby diagrams are drawn in Figure 22, connecting all these parameters according to Figure 9, Figure 14 and Figure 19.

Some factors that could affect the degradation mechanisms for the PPA/40CCF composite and, accordingly, its wear resistance, are presented below:

- CCFs were fractured under the dry-friction conditions due to the action of the shear load transmitted from the rotating counterfaces. On the one hand, their contribution was obligatory for ensuring the “volumetric” strength properties, while the “surface” tribological characteristics were determined by the interaction with the pressing and rotating counterface. The localization of strains in the surface layer with the high content of CCFs was accompanied by their fragmentation (fracturing), but the insufficient polymer content did not contribute to the formation of a mixing (ratcheting) layer reinforced with fractured CCFs (the so-called “tribological layer”). However, CCFs uniformly reinforced the wear track surface even without mixing, so a significant number of high-strength fibers effectively carried the *P* load. Nevertheless, a typical tribological layer was not formed [20].
- The chemical interaction between the counterfaces and the polymer matrix apparently could be the reason that the CoF level was lower by two times for the ceramics than that for the steel under the dry-friction conditions. However, this phenomenon could not have a decisive effect on the WR values, which were close for both counterparts at the *P* load of 180 N.
- The *Ra* roughness on the counterface surfaces also had to be taken into account under the dry-friction conditions. For the “ball-on-disk” scheme, the CoF levels were similar for the ceramic and steel counterparts, while the WR values were multiple times lower in the first case. According to the authors, asperities on the counterface surfaces did not “cling” to protruding CCFs, as they did on the “non-renewable” surface in the linear contacts. In these cases, the aspect of retaining CCFs through the polymer matrix was important in addition to providing the load-bearing capacity.
- The ratio of CCFs to the polymer matrix greatly affected the obtained results, since CCFs were characterized by the high mechanical properties, while PPA could locally withstand stresses many times greater than those caused by the applied *P* loads. As a result, the polymer matrix can be fractured, chipped, abrasively scratched (wear out), and plastically flow in the sliding direction due to the action of fractured CCFs.
- The counterface temperatures had a great effect, primarily of the ceramic one due to its low thermal conductivity. Accelerating the *V* sliding speed almost did not influence the CoF levels, while varying the *P* load caused their noticeable changes. At the same time, the WR values varied in approximately the same manner (Figure 22). Thus, the counterface temperature exerted minor influence on the WR values upon testing at the applied load–speed parameters.
- The role of adhesion should be stressed (based on the data shown in Figure 4), despite this factor not being varied and/or assessed in this study. The reason is the fact that the strength properties were significantly higher with great interfacial adhesion. In addition, an important condition for improving wear resistance is the retention of fractured CCFs by the polymer matrix in order to avoid micro-abrasive effects in the tribological contacts. The presented wear track surfaces did not contain any (micro)grooves oriented in the sliding direction. According to the authors, this phenomenon (along with high strength) contributed to the improvement of wear resistance of the PPA/40CCF composite.

**Figure 23:**
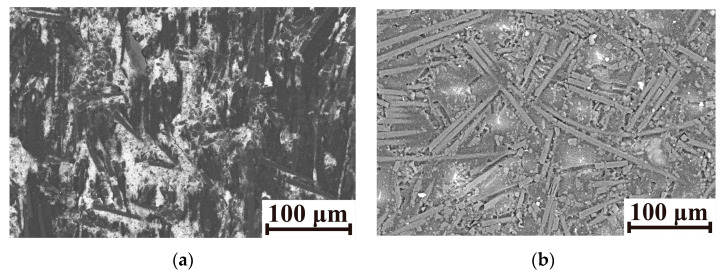

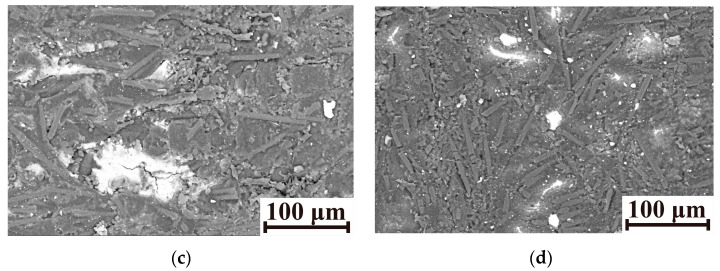
SEM micrographs of the wear track surfaces after the tribological tests in the linear contacts against the steel (**a**,**b**) and ceramic (**c**,**d**) counterfaces under the dry-friction (**a**,**c**) and oil-lubricated (**b**,**d**) conditions at the *P* load of 60 N and the *V* sliding speed of 0.3 m/s.

Based on the above, a question arose about the wear mechanism that was characteristic of the PPA/40CCF composite under the employed tribological conditions. To clarify this phenomenon, SEM micrographs of its wear track surfaces are shown in Figure 23 for the linear tribological contacts. After testing against the steel counterface under the dry-friction conditions, it was rough due to the adhesive interaction of the steel and the polymer matrix at the CoF level of 0.23 and the WR value of ~2.3 × 10^−6^ mm^3^/N·m. This fact was in good agreement with the results shown in Figure 6, according to which neat PPA was characterized by the significant increase in the WR value and the adhering of debris on the counterfaces as the *P* load was enhanced under the dry-friction conditions. When testing against the (more inert) ceramic counterface, the CoF level decreased down to ~0.13 and the WR value reduced by more than half. In addition, signs of local fracture of the polymer matrix were visible on the wear track surface. The authors tended to interpret this fact as the fatigue wear mechanism. According to Figure 23c, the polymer matrix locally cracked/chipped with satisfactory interfacial adhesion. It should be stressed that the suggested hypothesis is the authors’ point of view, and deeper studies are required in the nearest future. 

The oil-lubricated environment primarily reduced the adhesive interaction of the tribological contact components. For this reason, their wear mechanism became similar, the WR values were comparable (Table 5), and the wear track surfaces were almost identical (Figure 23b,d). So, the presence of an oil film additionally ensured the redistribution of the *P* load, lowering the WR values multiple times down to 0.15 × 10^−6^ and 0.55 × 10^−6^ mm^3^/N·m for the ceramic and steel counterfaces, respectively.

The composites investigated in this study (primarily the PPA/40CCF one) could be classified as materials suitable for heavy loaded friction units, since they showed a high shear strength of more than 350 MPa at a fiber content of 40 wt.%. This suggestion was ensured by the following set of factors: the great interfacial adhesion; the high both filling level; and the AR value (due to their fragmentation and dispersed strengthening of the wear track surface). At the same time, the high CoF level for neat PPA was fully compensated by the improved strength of the surface layer, when it was possible to both effectively implement its running-in and ensure an advanced load-bearing capacity.

## 5. Conclusions

Based on the determined mechanical properties and tribological characteristics of the commercially available PPA-based composites loaded with reinforced fibers, the following conclusions were drawn:

1. Under the applied test conditions, the PPA-based composite reinforced with 40 wt.% CCFs at AR~1000 possessed the best mechanical properties and tribological characteristics. Among other things, the reason was the dispersed hardening/reinforcement of the friction surfaces with fragmented (fractured) CCFs up to 100 μm long. In the linear tribological contact against the steel counterface at the *P* load of 60–180 N, their wear resistance was improved (WR ~ (2.0–3.5) × 10^−6^ mm^3^/N·m) even at the relatively high CoF level of ~0.23. Replacing the steel counterface with the ceramic one made it possible to reduce the adhesive interaction of the tribological contact components and decrease the WR values by more than half.
2. In the linear contact under the dry friction conditions at the *P* load of 60–180 N, the PPA/40CCF composite was characterized by the smaller scatter of the WR values when tested against the steel counterface than that for the ceramic one, with the approximately identical range of the CoF levels. In both cases, the friction surfaces were reinforced with fragmented CCFs. Thus, the WR values were determined to a greater extent by the load-bearing capacity of the composite rather than by the chemical interaction of the tribological contact components under such conditions.
3. When varying the *V* sliding speed at the constant *P* load in the linear tribological contact, the greater scatters of both CoF levels and WR values were evident for the PPA/40CCF composite, which was associated with significant heating of the ceramic counterface compared to the steel one, for which effective heat dissipation was realized. However, the WR values decreased when tested against the ceramic counterface as the *P* load increased. This phenomenon could be caused by the shorter interaction (due to the higher *V* sliding speed) of asperities on its surface and the additional thermally induced smoothing of the wear tracks on the PPA/40CCF composite.
4. In the linear tribological contact, similar changing kinetics in the ceramic counterface temperatures could be caused by enhancing both the *P* load and the *V* sliding speed. However, the increase in the *P* load from 60 up to 180 N enhanced the WR values to a noticeably greater extent than the increase in the *V* sliding speed. This fact means that the PPA/40CCF composite could maintain the WR values even upon the softening of the polymer matrix (the measured temperatures of the counterface yielded only underestimated integral values).
5. In both point and linear tribological contacts under the oil-lubricated conditions, the CoF levels and the WR values decreased (as expected). Therefore, such friction units could be recommended for practical implementation whenever possible.
6. One of the reasons for improving the tribological characteristics of the PPA/40CCF composite should be considered the fatigue wear mechanism, which was facilitated by a i) high filling degree; ii) strong interfacial adhesion; and iii) great AR value for CCFs.
7. As a practical aspect of the performed study, 2D and 3D Ashby diagrams should be recommended, connecting the key tribological characteristics of the studied materials in the linear tribological contacts under the applied load–speed conditions.

## Figures and Tables

**Table 1:** Functional properties and cost of PPA and some other HPPs for comparison [11].

Properties	PA12	PPA	PPS	PEEK
Density, g/cm^3^	1.04	1.18	1.35	1.32
Melting point, °C	180	310–330	280–290	365–375
Glass transition temperature, °C	40–50	121–138	90	150–160
Heat deformation temperature, °C	40–50	120	110	152
Modulus of elasticity, GPa	1.4	2.5–3.5	2.5	4.5
Tensile strength, MPa	50–60	90	75	90–100
Elongation at break (%)	300	6	15	20
Cost ($/kg)	9	5.2	7.5	65

**Table 2:** The grades and the key characteristics of neat PPA and PPA-based composites.

No.	Grade	Key Characteristics	Approximate Fiber Length/Diameter, μm *	Manufacturer	Country	Designation
1	N100	Neat PPA with a melting point of 320 °C and a glass transition temperature of ~123 °C; used as a matrix material for the fabrication of the composites.	-	Zhejiang NHU Comp. Ltd. (Shaoxing, China)	China	Neat PPA
2	KY-H90C30	PPA reinforced with 30 wt.% SCFs.	2000/10	Xiamen keyuan Plastic Co., Ltd. (Xiamen, China)	China	PPA/30SCF
3	PPA-NA-LGF40	PPA reinforced with 40 wt.% continuous CFs (CCFs); characterized by improved strength properties.	2000/10	Xiamen LFT Composite plastic Co., Ltd. (Xiamen, China)	China	PPA/40CCF
4	Amodel AS-1133 HS	PPA reinforced with 30 wt.% SGFs; heat stabilized.	8000/8	Solvay (Brussels, Belgium)	Belgium	PPA/30SGF
5	PPA-GFs-AF	PPA reinforced with 50 wt.% continuous GFs (CGFs); characterized by improved strength properties at a relatively low cost.	8000/18	DKM Engineering, Ekaterinburg, Russia/ISPMS SB RAS. Tomsk, Russia	Russia	PPA/50CGF

* According to the manufacturer.

**Table 3:** Physical and mechanical properties of neat PPA and PPA-based composites.

No.	Properties	Neat PPA	PPA/30SGF	PPA/30SCF	PPA/40CCF	PPA/50CGF
1	Density, g/cm^3^	1.20	1.45	1.59	1.31	1.58
2	Calculated density (relative to neat PPA)	1.00	1.23	1.35	1.12	1.16
3	Fiber content, vol. %	–	17.4%	24.8%	30%	31.6%
4	Approximate fiber length/diameter, μm	–	2000/10	2000/10	8000/8	8000/18
5	Melt flow rate, g/10 min (275 °C/2.16 kg)	49.5	n/a	n/a	n/a	n/a
6	Water absorption in 24 h, %	0.18 ± 0.03	0.22 ± 0.03	0.18 ± 0.02	0.22 ± 0.03	0.24 ± 0.04
7	Elastic modulus *, GPa	3.31 ± 0.09	9.01 ± 0.28	12.79 ± 0.56	33.67 ± 1.85	17.65 ± 0.55
8	Ultimate tensile strength *, MPa	97.72 ± 6.91	113.14 ± 9.70	142.72 ± 12.47	240.00 ± 3.00	207.00 ± 1.00
9	Flexural strength *, MPa	147.6 ± 16.8	172.2 ± 13.5	250.3 ± 18.6	394.0 ± 11.0	264.0 ± 6.0
10	Charpy impact strength *, kJ/m^2^	12.3 ± 3.1	21.6 ± 4.1	44.5 ± 7.3	55.8 ± 4.5	51.5 ± 2.9
11	Shore D hardness *	79.4 ± 0.3	76.7 ± 0.6	77.7 ± 0.5	82.1 ± 1.2	80.5 ± 1.3

* at room temperature.

**Table 4:** The tribological characteristics of neat PPA and the PPA-based composites; the steel counterface, the “block-on-ring” scheme.

No.	Composite	CoF			WR, mm^3^/N·m, 10^−6^			Temperature, °C		
		60 N	120 N	180 N	60 N	120 N	180 N	60 N	120 N	180 N
1	Neat PPA	0.45 ± 0.03	0.45 ± 0.04	0.57 ± 0.09	44.85 ± 1.1	55.04 ± 0.79	57.84 ± 3.98	32 ± 1	45 ± 5	70 ± 16
2	PPA/30SCF	0.26 ± 0.03	0.30 ± 0.02	0.30 ± 0.01	12.74 ± 1.45	16.64 ± 1.66	22.13 ± 0.44	26 ± 1	26 ± 2	37 ± 2
3	PPA/40CCF	0.23 ± 0.02	0.23 ± 0.01	0.24 ± 0.01	2.33 ± 0.31	2.41 ± 0.14	3.47 ± 0.16	28 ± 1	23 ± 1	27 ± 1
4	PPA/30SGF	0.28 ± 0.04	0.32 ± 0.02	0.36 ± 0.01	36.62 ± 2.04	37.57 ± 0.66	56.75 ± 4.77	27 ± 1	29 ± 1	50 ± 4
5	PPA/50CGF	0.32 ± 0.03	0.33 ± 0.01	0.37 ± 0.01	3.36 ± 0.31	3.77 ± 0.24	3.81 ± 0.22	28 ± 1	29 ± 1	31 ± 3

**Table 5:** The tribological characteristics of the PPA/40CCF composite; the steel and ceramic counterfaces, the “block-on-ring” scheme; load variation.

No.	Composite; Test Conditions	CoF			WR, mm^3^/N·m, 10^−6^			Temperature, °C		
		60 N	120 N	180 N	60 N	120 N	180 N	60 N	120 N	180 N
1	PPA/40CCF; metal, dry	0.23 ± 0.02	0.23 ± 0.01	0.24 ± 0.01	2.33 ± 0.31	2.41 ± 0.14	3.47 ± 0.16	28 ± 1	23 ± 1	27 ± 1
2	PPA/40CCF; ceramic, dry	0.13 ± 0.02	0.14 ± 0.01	0.12 ± 0.01	1.07 ± 0.20	1.59 ± 0.36	3.05 ± 0.38	40 ± 2	47 ± 2	61 ± 3
3	PPA/40CCF; metal, oil	0.05 ± 0.02	0.08 ± 0.01	0.09 ± 0.01	0.55 ± 0.07	0.29 ± 0.04	0.35 ± 0.04	25 ± 1	28 ± 2	35 ± 2
4	PPA/40CCF; ceramics, oil	0.03 ± 0.02	0.07 ± 0.01	0.08 ± 0.01	0.15 ± 0.02	0.19 ± 0.04	0.24 ± 0.02	25 ± 1	32 ± 1	37 ± 1

**Table 6:** The tribological characteristics of the PPA/40CCF composite; the steel and ceramic counterfaces, the “block-on-ring” scheme; sliding speed variation.

No.	Composite	CoF			WR, mm^3^/N·m, 10^−6^			Temperature, °C		
		0.3 m/s	0.5 m/s	0.7 m/s	0.3 m/s	0.5 m/s	0.7 m/s	0.3 m/s	0.5 m/s	0.7 m/s
1	PPA/40CCF; steel, dry	0.23 ± 0.02	0.17 ± 0.01	0.18 ± 0.02	2.33 ± 0.31	1.58 ± 0.23	2.63 ± 0.28	28 ± 1	24 ± 1	25 ± 1
2	PPA/40CCF; ceramic, dry	0.13 ± 0.02	0.1 ± 0.02	0.06 ± 0.01	1.07 ± 0.2	0.99 ± 0.18	0.37 ± 0.05	40 ± 2	47 ± 2	49 ± 4
3	PPA/40CCF; steel, oil	0.05 ± 0.02	0.009 ± 0.01	0.008 ± 0.01	0.55 ± 0.07	0.019 ± 0.01	0.022 ± 0.01	25 ± 1	27 ± 1	26 ± 1
4	PPA/40CCF; ceramics, oil	0.03 ± 0.02	0.017 ± 0.01	0.014 ± 0.01	0.15 ± 0.02	0.09 ± 0.01	0.05 ± 0.01	25 ± 1	27 ± 1	30 ± 1

**Table 7:** The tribological characteristics of the PPA/40CCF composite; the steel and ceramic counterfaces, the “ball-on-disk” scheme.

Composite	CoF				WR, mm^3^/N·m, 10^−6^			
	Steel		Ceramic		Steel		Ceramic	
	Dry	Oil	Dry	Oil	Dry	Oil	Dry	Oil
PPA/40CCF	0.270 ± 0.006	0.050 ± 0.003	0.240 ± 0.010	0.060 ± 0.002	18.9 ± 6.4	0.3 ± 0.1	5.4 ± 2.2	1.5 ± 0.5

## Data Availability

The data presented in this study are available upon request from the corresponding author due to the privacy reason.
